# Healthy Eating beyond Whole Grains—Insight on Associations between Diet Quality and Arterial Stiffness in the Brisighella Heart Study Cohort

**DOI:** 10.3390/nu16162792

**Published:** 2024-08-21

**Authors:** Marina Giovannini, Federica Fogacci, Sergio D’Addato, Elisa Grandi, Claudio Borghi, Arrigo F. G. Cicero

**Affiliations:** 1 Hypertension and Atherosclerosis Research Group, Medical and Surgical Sciences Department, Alma Mater Studiorum University of Bologna, 40138 Bologna, Italy; marina.giovannini3@unibo.it (M.G.); sergio.daddato@unibo.it (S.D.); elisa.grandi@unibo.it (E.G.); claudio.borghi@unibo.it (C.B.); arrigo.cicero@unibo.it (A.F.G.C.); 2 Cardiovascular Medicine Unit, IRCCS Azienda Ospedaliero-Universitaria di Bologna, 40138 Bologna, Italy

**Keywords:** healthy eating, heathy diet, whole grain foods, blood pressure, arterial stiffness, pulse wave velocity

## Abstract

Although whole grains have well-recognized protective effects against the development of cardiometabolic diseases, whole grain foods are poorly consumed by the general population. The aim of our study was to establish, at a population level, the vascular impact of a low intake of whole grain foods. From the initial cohort of the Brisighella Heart Study, we identified a population sample of 1503 individuals—including 720 men (47.9%) and 783 women (52.1%)—who overall largely consumed refined grain products. Diet quality was estimated by the Short Healthy Eating Index (sHEI), and women were found to have an eating pattern that was overall healthier than men (44.1 ± 8.5 vs. 36.3 ± 8.1, *p* < 0.001). The development of an age- and blood pressure (BP)-adjusted multiple linear regression model found that carotid–femoral pulse wave velocity (cfPWV) was significantly predicted by the estimated glomerular filtration rate (eGFR, B = −0.148, 95% Confidence Interval (CI) −0.259–−0.038, *p* < 0.001), serum uric acid (SUA, B = 0.220, 95%CI 0.095–0.320, *p* = 0.001) and sHEI (B = −0.231, 95%CI −327–−0.089, *p* < 0.001) in men, and by eGFR (B = −0.152, 95%CI −0.266–−0.052, *p* < 0.001), body mass index (BMI, B = 0.174, 95%CI 0.111–0.331, *p* = 0.002), SUA (B = 0.278, 95%CI 0.158–0.354, *p* < 0.001) and sHEI (B = −0.218, 95%CI −308–−0.115, *p* < 0.001) in women. Ultimately, a low sHEI score was a significant predictor of arterial stiffness also in a population cohort with a high consumption of refined grain products.

## 1. Introduction

Mounting evidence supports the beneficial effects of a high intake of whole grains in reducing the risk of non-communicable diseases, such as cardiovascular diseases, type 2 diabetes, and certain types of cancer [1,2]. These grains encompass the entire grain seed, including the bran, germ and endosperm, offering a rich source of essential nutrients, dietary fiber, and phytochemicals [3]. The dietary fiber found in whole grains aids in digestion, promotes satiety, and helps regulate blood sugar levels, reducing the risk of obesity, insulin resistance, and type 2 diabetes [4]. In addition, they support a healthy gut microbiome, contributing to improved gastrointestinal health and overall well-being [5]. Whole grains are also abundant sources of vitamins, minerals and phytochemicals, including antioxidants and polyphenols, with anti-inflammatory and disease-fighting properties [6]. These bioactive compounds play a crucial role in mitigating oxidative stress, combating inflammation and enhancing immune function, reducing the risk of chronic diseases and promoting longevity [1,6].

Incorporating whole grains into the diet has also been associated with improved cardiovascular health. The regular consumption of whole grains has been linked to lower levels of low-density lipoprotein cholesterol (LDL-C), triglycerides (TG), and blood pressure (BP), leading to a reduced risk of cardiovascular diseases such as heart attack and stroke [7,8]. The bioactive compounds—such as lignans and phytosterols—contained in the whole grains further contribute to cardiovascular protection through their cholesterol-lowering and anti-inflammatory effects [8].

Despite this evidence, whole grain foods are still scarcely consumed in the general population [9]. In this context, the aim of our study was to establish, at a population level, the vascular impact of a low intake of whole grain foods.

## 2. Materials and Methods

### 2.1. Study Participants and Design

The Brisighella Heart Study (BHS) is an ongoing prospective population-based cohort study established in 1972 and enrolling 2939 healthy Caucasian adults (1491 men and 1448 women) with no prior heart disease. BHS participants were recruited from the village of Brisighella and selected to be representative of the general population in Italy.

The BHS adheres to the ethical principles of the Declaration of Helsinki. Study procedures have been approved by the Ethics Committee of the IRCCS (Istituto di Ricovero e Cura a Carattere Scientifico) University Hospital of Bologna (Code: BrixFollow-up_1972-2024). Participants undergo clinical evaluations every four years, with high follow-up rates. Over the time, mortality, morbidity, and the occurrence of major cardiovascular risk factors have been collected. Informed consent was obtained from each participant at entry and updated at each examination.

#### Inclusion and Exclusion Criteria

**Figure 1:**
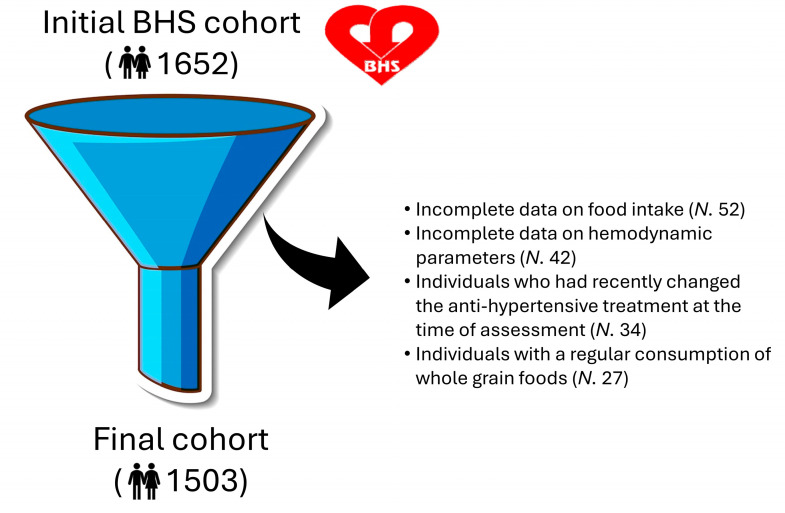
Flowchart of the study population.

Of the 1652 individuals involved in the last population survey, BHS participants with incomplete information on food intake (*N.* 52) and/or hemodynamic parameters (*N.* 42) and those who had recently changed the antihypertensive treatment at the time of assessment (*N.* 34) were excluded. Individuals with a regular consumption of whole grain foods (*N.* 27) were also excluded from the analysis (Figure 1).

Our final cohort included 1503 individuals, of whom 720 were men (47.9%) and 783 were women (52.1%)

### 2.2. Covariate Ascertainment

Medical history, laboratory data, anthropometric and hemodynamic measurements were ascertained as described in earlier publications [10]. In summary, detailed personal and family medical histories, lifestyle and dietary habits, smoking status and pharmacological treatments were recorded. Fasting blood samples were collected, and physical examinations (including anthropometric measurements, resting blood pressure and heart rate) and standard 12-lead electrocardiograms were performed [10].

#### 2.2.1. Clinical Assessment

The height was measured in centimeters (cm), asking to participants to stand erect, with bare foot together and eyes directed straight ahead. Weight was measured twice, and the average of these two measures was used. Body mass index (BMI) was calculated as weight in kilograms divided by height in meters squared (kg/m^2^). Waist circumference was measured on the horizontal plane midway between the lowest ribs and the iliac crest. The hip circumference was measured at the widest part of the hips. The waist-to-hip ratio was also calculated. The index of central obesity (ICO) was calculated by the ratio of waist circumference and height [11].

The Dietary Quality Index (DQI) was used to quantify the overall quality of individuals’ usual food intake in the year before the assessment [12]. This composite measure was calculated using a semi-quantitative food frequency questionnaire containing 18 food items grouped in 3 categories [13,14]. Moreover, the Short Healthy Eating Index (sHEI) was computed as the sum of the frequency consumption of food groups [15].

#### 2.2.2. Hemodynamic Evaluation

Systolic BP (SBP) and diastolic BP (DBP) were measured 3 times 60 s apart using an automated electronic sphygmomanometer with the subject in the seated position for 5 min. The mean of the three BP readings (for both SBP and DBP) was used as study variables and to compute pulse pressure (PP) and mean arterial pressure (MAP) [16]. PP was calculated as the difference between SBP and DBP, and MAP was calculated as DBP + 1/3 (SBP–DBP). Consistently with the latest international guidelines [16], the presence of hypertension was defined as SBP ≥ 140 mmHg and/or DBP ≥ 90 mmHg, or self-reported use of antihypertensive drugs. Arterial stiffness parameters were noninvasively evaluated using the Vicorder^®^ apparatus (Skidmore Medical Ltd., Bristol, UK), which is a validated operator-independent cuff-based device previously used in other epidemiological studies [17,18]. The Augmentation Index (AIx) was obtained with a brachial cuff placed at the right arm, through the BP waveform analysis, and calculated as the ratio of the pressure increment caused by the reflected wave (augmented pressure) to the PP [19]. Additional central hemodynamic parameters were derived from brachial BP waveforms self-calibrated to brachial SBP and DBP, following a previously described brachial-to-aortic transfer function [20,21]. Carotid–femoral pulse wave velocity (cfPWV) was measured with a simultaneous measurement of carotid and femoral BP through a neckpad containing a photoplethysmographic detector placed around the neck and a normal cuff positioned around the thigh [22].

#### 2.2.3. Laboratory Assessment

A serum panel that included glucose, total cholesterol (TC), TG, high-density lipoprotein cholesterol (HDL-C), apolipoprotein B (Apo B), apolipoprotein AI (Apo AI), lipoprotein(a) (Lp(a)), aspartate transaminase (AST), alanine transaminase (ALT), gamma-glutamyl transferase (gGT), creatinine and uric acid (SUA) was drawn after a 12 h fasting. All laboratory parameters were ascertained with standardized methods by trained personnel [23]. LDL-C was calculated with the Friedewald equation (LDL-C = TC − HDL-C − (TG/5)) [24]. The glomerular filtration rate (eGFR) was estimated by the Chronic Kidney Disease Epidemiology Collaboration (CKD-EPI) formula [25].

### 2.3. Statistical Methods

The distribution of cohort characteristics was determined within each sex, and a full descriptive analysis was run on the dataset. All continuous variables were checked for their distributions using the Kolmogorov–Smirnov normality test. Outcomes of clinical evaluations were compared between sexes with Student’s *t*-test. Variables that were not normally distributed were log-transformed before going on with the analyses. Categorical variables distribution was compared through the Chi-square test.

We carried out a univariate analysis in order to identify variables associated with cfPWV (*p* < 0.20), which were to be included in a regression model together with those expected to be relevant according with the current literature independently of their results in the univatiate analysis.

Then, an age- and BP-adjusted linear multiple step-wise regression model was developed to predict cfPWV as a function of sex, BMI, smoking habit, LDL-C, TG, glucose, SUA, eGFR and sHEI, using smoking habit as an independent variable. The analysis was then repeated by sex. All tests were performed using SPSS 27.0 for Windows (IBM Corporation, Armonk, NY, USA). A significance level of 0.05 was considered to be statistically significant for all tests.

## 3. Results

Our cohort included 783 women (mean age 58.2 ± 15.8) and 720 men (mean age 58.2 ± 15.6 years). Among women, 19% were active smokers, 18% were previous smokers and 63% were never smokers; among men, 18% were active smokers, 19% were previous smokers, and 63% were never smokers (chi-square: 54.394, *p* < 0.001).

The main anthropometric and hemodynamic characteristics of the BHS participants are shown by sex in Table 1. There were no differences in age, hip circumference, ICO, SBP, aortic BP and PWV (Table 1). However, the WC, BMI, waist/hip ratio, HR, DBP and MAP were significantly lower (*p* < 0.001) among women. Women were also more likely to have a higher PP, PP index, aortic PP and augmentation index than men (*p* < 0.001).

Consistently, the prevalence of hypertension (34% vs. 29%, *p* = 0.023), obesity (19% vs. 16%, *p* = 0.038) and dyslipidemia (38% vs. 32%, *p* = 0.001) was significantly higher in men than in women.

Table 2 shows significant differences in laboratory parameters between the study groups.

Even if Apo B was similar among groups, the TC, LDL-C, Lp(a) and LDL-C/Apo B ratio were higher among women. Men were also more likely to have lower HDL-C and Apo AI. On the contrary, fasting glucose, TG, AST, ALT, gamma-GT, SUA, creatinine, eGFR and CPK were higher among men than women.

**Figure 2:**
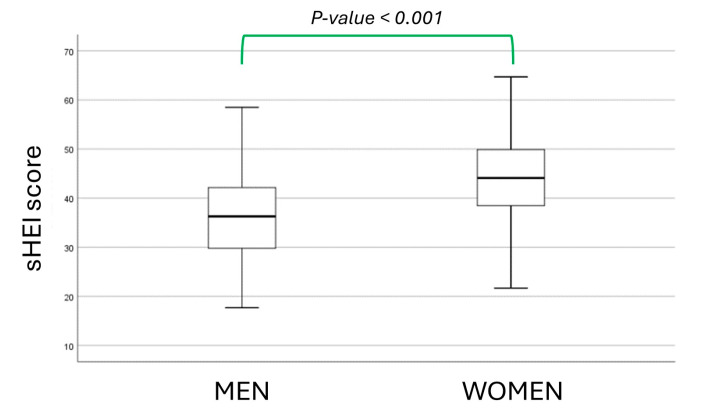
sHEI score distribution in men and women.

The sHEI score was 36.3 ± 8.1 in men and 44.1 ± 8.5 in women (*p* < 0.001 for the between-sex comparison; see Figure 2).

Men reported eating more bread (*p* < 0.001), pasta (*p* < 0.001), high-fat meat (*p* < 0.001) and alcoholic drinks (*p* < 0.001) than women, who consumed a higher amount of green vegetables (*p* < 0.001), other vegetables (*p* < 0.001), fruits (*p* < 0.001), dairy products (*p* < 0.001), extra virgin olive oil (*p* < 0.001) and other vegetable oils (*p* < 0.001), seed oils (*p* < 0.001), coffee and tea (*p* = 0.013), salami, bony fishes (*p* = 0.014) and nuts (*p* < 0.001). The use of sugar (*p* = 0.769), soft drinks (*p* = 0.063), low-fat meat (*p* = 0.329), ham (*p* = 0.455), cheese (*p* = 0.362), animal fats (*p* = 0.947), crustaceans and mollusks (*p* = 0.516) and legumes (*p* = 0.105) were similar in the groups (Supplementary Figures S1–S3).

In the whole sample, an age- and BP-adjusted linear multiple step-wise regression model showed that the main predictors of cfPWV were sex (B = 0.381, 95%CI = 0.231 to 0.522, *p* < 0.001), eGFR (B = −0.151, 95%CI = −0.245 to 0.043, *p* < 0.001), SUA (B = 0.246, 95%CI = 0.123 to 0.338, *p* < 0.001), sHEI (B = −0.241, 95%CI = −0.315 to 0.103, *p* < 0.001), and smoking (B = 0.098, 95%CI = 0.011 to 0.131, *p* = 0.019). BMI, fasting plasma glucose, LDL-C, and TG were not statistically associated with cfPWV in the whole cohort.

After adjustment for age and BP, among men, significant predictors arterial stiffness were low eGFR, low sHEI score and high levels of SUA (Table 3). Among women, increased arterial stiffness was significantly associated with higher BMI, higher levels of SUA, low eGFR and low sHEI score (Table 3).

## 4. Discussion

Diet is the cornerstone of preventing cardiovascular disease and improving BP control [26,27].

In theory, it is assumed that in Mediterranean countries, there is a high consumption of whole grain foods [28,29]. However, the intensification of the grain culture and the industrialization of its processing led to a significant reduction in the consumption of whole grain foods [30,31]. Moreover, especially in rural areas and among elderly people, whole foods are little appreciated because they are historically associated with periods of poverty, while bread and other foods produced with refined flours have become popular in periods of economic boom [31,32].

In our rural population cohort of individuals with a low intake of whole grain foods, an age- and BP-adjusted linear multiple step-wise regression model showed that the main predictors of cfPWV were sex, eGFR, SUA, sHEI, and smoking. BMI, fasting plasma glucose, LDL-C, and TG were not statistically associated with cfPWV in the whole cohort. These data have to be put in context.

In fact, in our cohort, women had an overall healthier dietary pattern than men, usually eating more vegetables and bony fish. On the contrary, men’s dietary habits included a greater amount of more carbohydrates and high-fat meat. Indeed, men were more likely to be overweight and have hypertension, dyslipidemia and higher levels of fasting glucose and liver enzymes. After adjustment for age and BP, arterial stiffness was significantly associated with low eGFR, low sHEI score and high levels of SUA among men, and with higher BMI, higher levels of SUA, low eGFR and low sHEI score in women.

It must be acknowledged that in the same population cohort, we have previously reported that the use of extra-virgin olive oil as raw seasoning and in cooking significantly predicted cfPWV after adjustment for age and SBP (relative risk (RR) = 0.84, 95% CI 0.67–0.94 vs. other fatty sources) [33]. In another analysis, we found that cfPWV was significantly higher in individuals who had a high dietary fructose intake, especially when it was associated with a high use of industrially sweetened beverages, among which fructose from both fruits and sugar-sweetened beverages was a significant predictor of cfPWV (B = 0.310, 95%CI 0.099–0.522, *p* = 0.004) [33]. On the other side, coffee intake was associated with improved peripheral and central BP values without any relevant effect on cfPWV [34].

According to the current observations, men had less healthy dietary habits than women as well as a higher prevalence of the main cardiovascular risk factors (i.e., overweight, hypertension and higher plasma levels of atherogenic lipoproteins). Although it may seem otherwise, our results do not contradict previous literature but rather allow us to critically analyze it. In epidemiological studies where their intake has been associated with positive clinical outcomes, data have always been adjusted by the total energy intake, since whole grain foods are a relevant source of energy. Therefore, in the general overweight/obese population, the definition of whole grain products as healthy foods should be reconsidered, emphasizing the need for controlled energy intake, and including the absolute amount of whole grain products as the main source of carbohydrates and lipids. Our data underscore the critical issue that an overall healthy diet is a good predictor of vascular health even in a population with a low intake of whole grain foods. This is especially relevant when energy restriction is necessary. It must also be recognized that in our study, a short dietary questionnaire aiming at evaluating the degree of healthy diet has been useful to detect which dietary components were associated with better vascular health in general population. Of course, our analysis does not aim at reducing the importance of whole grain foods in the context of an energy-controlled healthy diet. Moreover, our study does have certain limitations. Firstly, in the BHS, food consumption data relied on self-reports, which may have led to inaccuracies. However, the dietary questionnaires were administered by trained personnel to enhance reporting quality. Secondly, some participants were receiving treatment with antihypertensive medications. To mitigate potential bias and ensure the reliability of the results, individuals who had recently changed antihypertensive medications at the time of assessment were excluded from the analysis. Then, our analysis was based on dietary data sampled in a single survey. From one side, we tried to reduce bias related to this limitation by personally administering the questionnaire to the enrolled subjects. On the other side, we are relatively sure that our historical cohort changes its dietary habit slowly. Lastly, the characteristics of the individuals in our cohort—including their rural background, sustained dietary habits over decades, genetic homogeneity and comprehensive availability of central BP and arterial stiffness data—may limit the comparability of our findings with those from other Italian and European cohorts.

Of course, our data are not contrasting the general indication to increase the whole grain foods quota in the diet in order to prevent cardiovascular diseases [35,36], but they show which could be the cardiovascular health determinants in subjects not assuming a relevant amount of whole grain foods in their everyday diet.

## 5. Conclusions

In conclusion, according to our observations, a low sHEI score was a significant predictor of arterial stiffness also in a population cohort with a high consumption of refined grain products.

## Figures and Tables

**Table 1:** Main anthropometric and hemodynamic measurements.

Parameters	Men (Mean ± SD)	Women (Mean ± SD)	*p*-Value
Age (years)	58.2 ± 15.6	58.2 ± 15.8	0.977
Waist Circumference (cm)	97.1 ± 11.3	87.9 ± 13.5	<0.001
Hip Circumference (cm)	100.9 ± 11.8	100.4 ± 3.2	0.468
BMI (kg/m^2^)	27.1 ± 3.9	26.3 ± 5.1	<0.001
Waist/Hip Ratio	0.95 ± 0.09	0.87 ± 0.09	<0.001
Index of Central Obesity	0.56 ± 0.07	0.56 ± 0.09	0.120
Heart Rate (bpm)	61.5 ± 11.4	66.3 ± 11.3	<0.001
SBP (mmHg)	140.8 ± 8.6	141.2 ± 12.8	0.705
DBP (mmHg)	75.4 ± 4.7	71.8 ± 4.5	<0.001
Pulse Pressure (mmHg)	65.4 ± 7.3	69.3 ± 9.7	<0.001
MAP (mmHg)	97.2 ± 11.5	94.9 ± 12.5	<0.001
Pulse Pressure Index	0.46 ± 0.06	0.48 ± 0.06	<0.001
Aortic BP (mmHg)	137.5 ± 8.6	138.3 ± 12.8	0.459
Aortic PP (mmHg)	62.1 ± 7.2	66.5 ± 9.6	<0.001
Augmentation Index	24.3 ± 8.8	26.7 ± 8.9	<0.001
Pulse Wave Velocity (m/s)	9.2 ± 2.2	9.1 ± 2.5	0.441

BMI = body mass index; BP = blood pressure; DBP = diastolic blood pressure; MAP = mean arterial pressure; SBP = systolic blood pressure; SD = standard deviation.

**Table 2:** Comparison between the two groups as regards laboratory data.

Parameters	Men (Mean ± SD)	Women (Mean ± SD)	*p*-Value
TC (mg/dL)	210.7 ± 40	220.2 ± 39.7	<0.001
TG (mg/dL)	126.8 ± 79.2	111.1 ± 60.2	<0.001
HDL-C (mg/dL)	48 ± 13.9	55.1 ± 15.6	<0.001
LDL-C (mg/dL)	137.8 ± 36.4	143 ± 37.1	0.006
Lp(a) (mg/dL)	20.1 ± 27.9	22.9 ± 30	0.021
Apo B (mg/dL)	90.3 ± 18.7	92.0 ± 21.9	0.099
Apo AI (mg/dL)	145.6 ± 24.1	160.8 ± 29.3	<0.001
LDL-C/Apo B	1.53 ± 0.31	1.57 ± 0.32	0.016
HDL-C/Apo AI	0.32 ± 0.06	0.34 ± 0.06	0.062
Fasting glucose (mg/dL)	98.2 ± 11	93.0 ± 17.9	<0.001
AST (U/L)	24.9 ± 8.7	22.2 ± 12	0.002
ALT (U/L)	28.6 ± 6.7	20.8 ± 11	<0.001
Gamma-GT (U/L)	33.6 ± 11.1	21.3 ± 12.8	<0.001
SUA (mg/dL)	5.9 ± 1.1	4.6 ± 1.2	<0.001
Creatinine (mg/dL)	1.14 ± 0.17	0.95 ± 0.16	<0.001
eGFR (mL/min)	72.7 ± 15.1	69 ± 15.7	<0.001
CPK (U/L)	147.1 ± 87.1	113.3 ± 72.8	<0.001

Apo AI = apolipoprotein AI; Apo B = apolipoprotein B; ALT = alanine aminotransferase; AST = aspartate aminotransferase; CPK = creatine phosphokinase; eGFR = estimated glomerular filtration rate; Gamma-GT = gamma-glutamyl transpeptidase; HDL-C = high-density lipoprotein cholesterol; LDL-C = low-density lipoprotein cholesterol; Lp(a) = lipoprotein(a); SD = standard deviation; SUA = serum uric acid; TC = total cholesterol; TG = triglycerides.

**Table 3:** Significant predictors of cfPWV in the subgroups after adjustment for BP and age (independent variables included: BMI, smoking habit, LDL-C, TG, glucose, SUA, eGFR and sHEI).

Sex	Predictors	Direction of the Prediction	B	95% Confidence Interval		*p*-Value
				Lower Limit	Upper Limit	
Men	eGFR	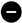	−0.148	−0.259	−0.038	<0.001
	SUA	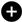	0.220	0.095	0.320	0.001
	sHEI	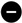	−0.231	−0.327	−0.089	<0.001
Women	BMI	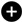	0.174	0.111	0.331	0.002
	eGFR	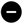	−0.152	−0.266	−0.052	<0.001
	SUA	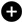	0.278	0.158	0.354	<0.001
	sHEI	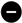	−0.218	−0.308	−0.115	<0.001

B = unstandardized coefficients; BMI = body mass index; eGFR = estimated glomerular filtration rate; sHEI = short healthy eating index; SUA = serum uric acid.

## Data Availability

Data are available by adequate request to the author with limitation related to the rules of local health system and based on the limitations reported in the protocol approved by the local Ethical Board.

## Supplementary Materials

The following supporting information can be downloaded at https://www.mdpi.com/article/10.3390/nu16162792/s1, Figure S1. Frequency of consumption of the main food items in men and women (* = *p* < 0.05 between sexes). Figure S2. Frequency of consumption of the main food items in men and women (* = *p* < 0.05 between sexes). Figure S3. Frequency of consumption of seasoning and cooking fats, fruits, sugar and coffee and tea in men and women (* = *p* < 0.05 between sexes).
