# Supplementary figures and images for: Healthy Eating beyond Whole Grains—Insight on Associations between Diet Quality and Arterial Stiffness in the Brisighella Heart Study Cohort
Source: Nutrients. 2024 Aug 21;16(16):2792. doi: 10.3390/nu16162792 (PMC11357628; doi:10.3390/nu16162792)

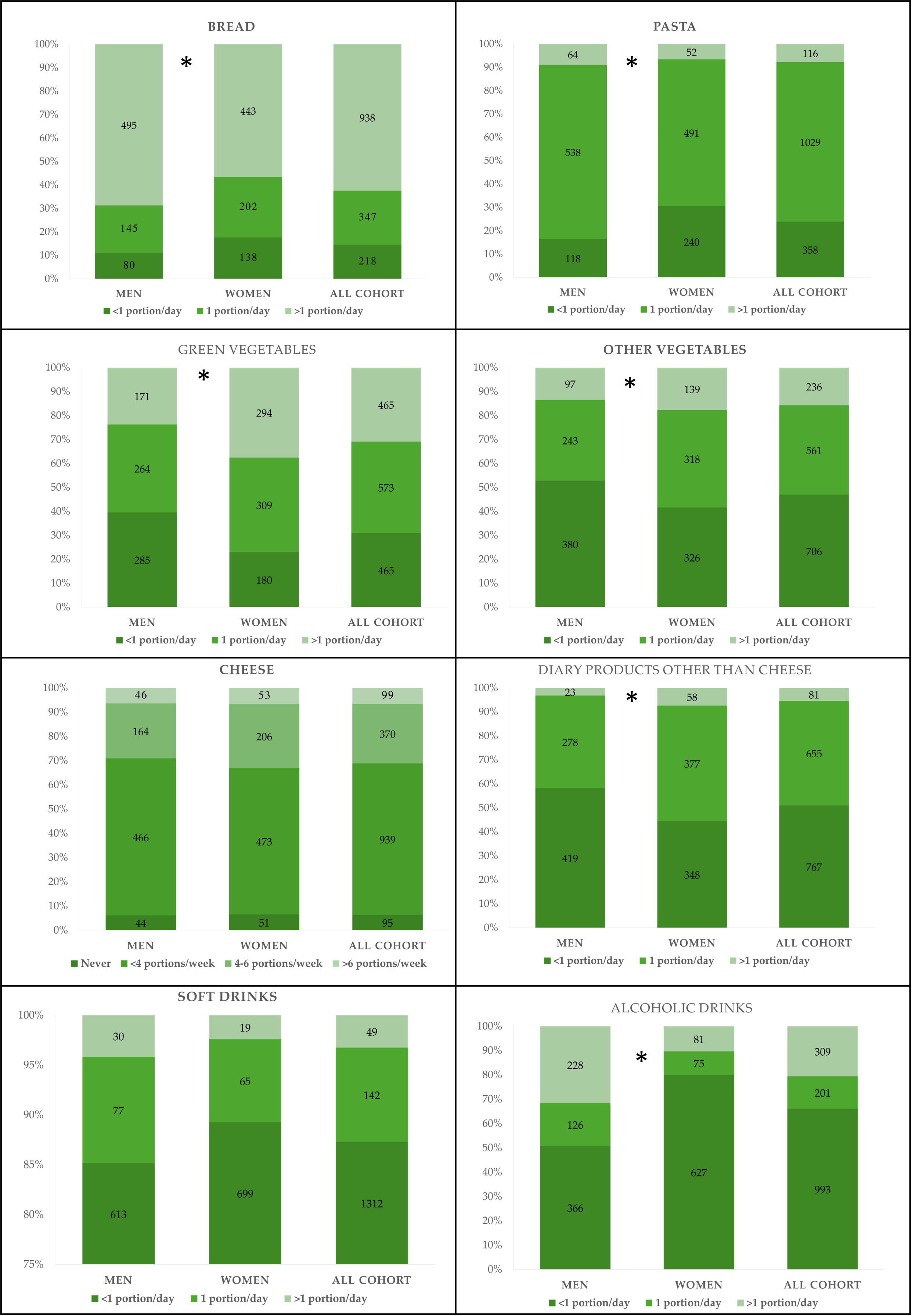

Supplement: Supplementary file 1 [file nutrients-16-02792-s001.zip › Figure 1_Suppl.png]

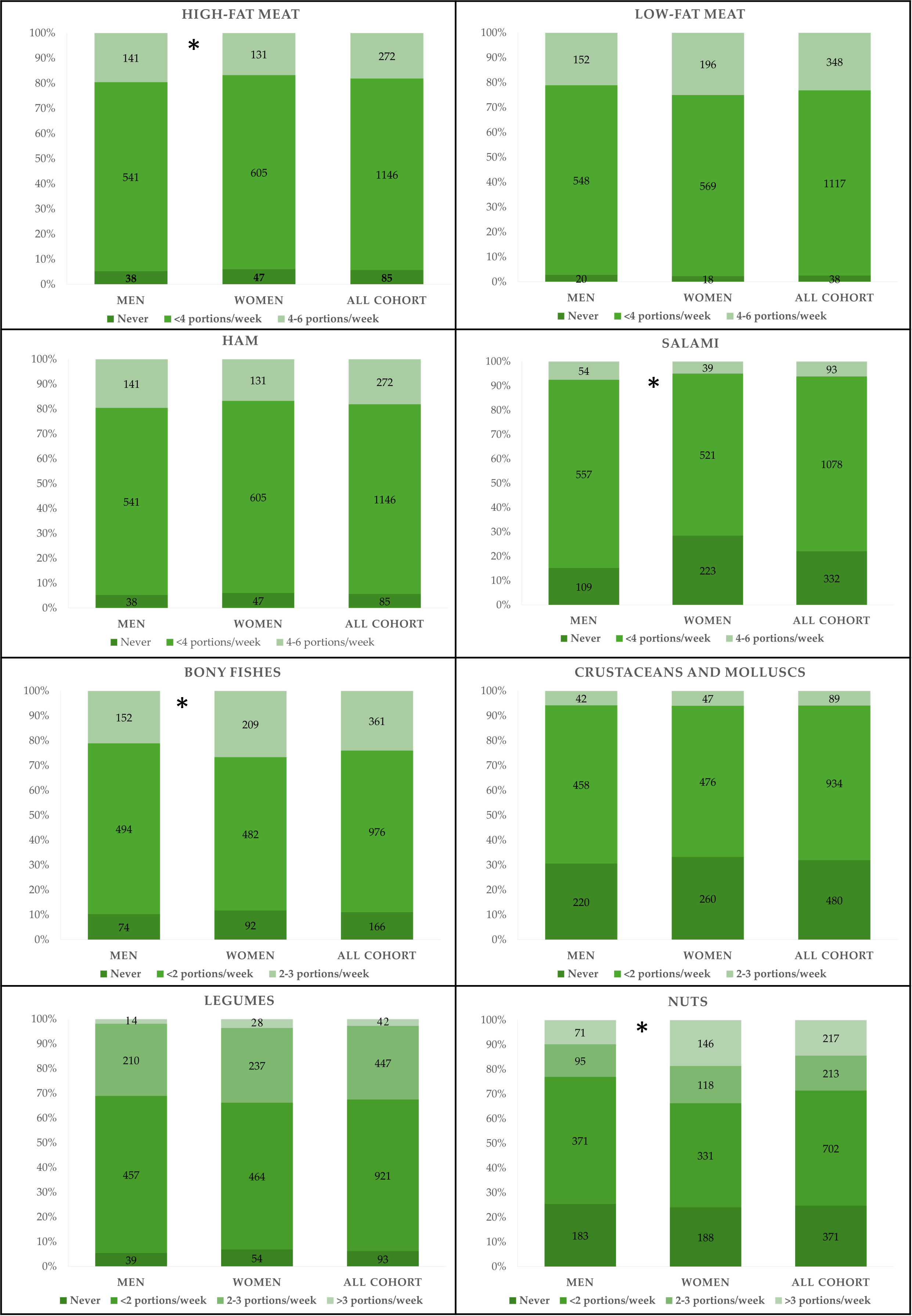

Supplement: Supplementary file 1 [file nutrients-16-02792-s001.zip › Figure 2_Suppl.png]

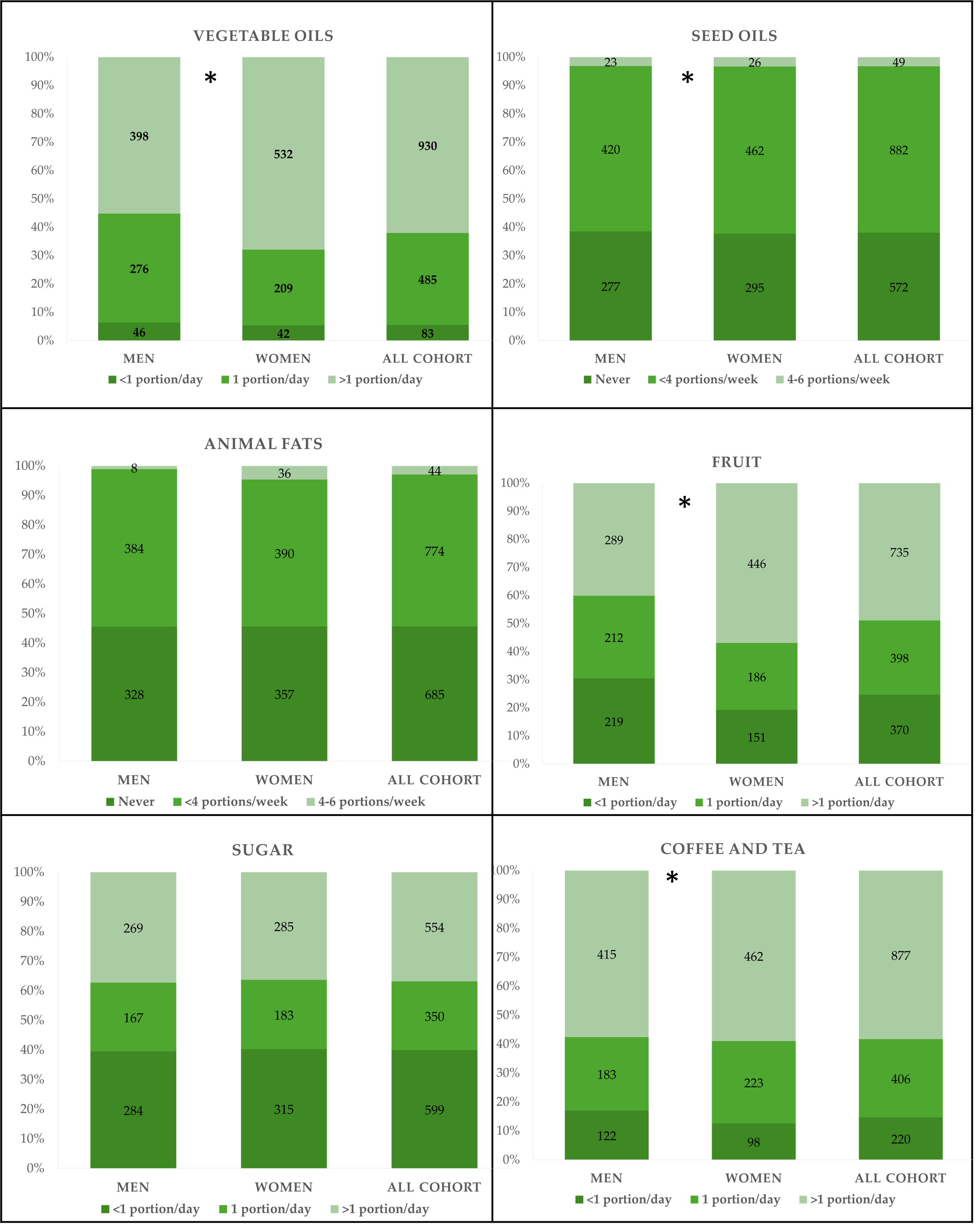

Supplement: Supplementary file 1 [file nutrients-16-02792-s001.zip › Figure 3_Suppl.png]
